# Genetic analysis of silique and seed traits in *Brassica juncea* (L.) Czern. under differential doses of nitrogen application

**DOI:** 10.1038/s41598-025-07758-0

**Published:** 2025-07-04

**Authors:** Javed Akhatar, Anna Goyal, Meenakshi Mittal, Heena Verma, Kaur Gurpreet, Beerpal Kaur, Surinder S. Banga, Chhaya Atri

**Affiliations:** Department of Plant Breeding and Genetics, Punjab Agricultural University, Ludhiana, Punjab 141004 India

**Keywords:** Rapeseed-mustard, Genome-wide association studies, Candidate genes, Pod shattering, RNA-seq, Rupture energy, Genetics, Molecular biology

## Abstract

Mustard, a major source of edible and industrial grade oils in the Indian subcontinent and various regions of Australia, Eastern Europe, and Canada, is also a protein resource for the animal feed industry. Silique and seed size are key traits for mustard improvement, but their inheritance mechanisms are not fully understood. We evaluated 92 inbred lines for silique length, seeds per silique, seed size, and rupture energy required to shatter a pod at three levels of nitrogen application in two crop seasons. Genotypes showed large phenotypic variations and a continuous distribution for all silique-related traits, suggesting a quantitative inheritance. Genotype × nitrogen interactions were significant for silique length, seeds per silique, and rupture energy. Association analysis identified 59 significant SNPs, whose annotations facilitated the prediction of 16 important genes underlying observed phenotypic variations. These genes are associated with silique formation (SHP2), grain formation (*BG1*, *BG4*), cell elongation (*BRI1*), grain filling (*AT1G12500*, *AT1G77610*, *SWEET1*, and *AT3G14410*), and silique shattering (*UBP15*, *CO*, *INDEHISCENT*, *AGAMOUS1*, *FRUITFULL*, and *SHP2*). RNA-seq data from 18 mustard genotypes revealed expression-level variations for identified candidate genes. Upregulation of *SHP2* for SPS and resistance to silique shattering was observed, while downregulation of *BG4* was observed in two genotypes with smallest seeds. This study provides valuable insight that may facilitate the marker-assisted selection (MAS) breeding for silique traits.

**Supplementary Information:**

The online version contains supplementary material available at 10.1038/s41598-025-07758-0.

## Introduction

Indian mustard (*Brassica juncea* (L.) Czern and Coss.) belongs to the rapeseed-mustard group of crops, which ranks among the most traded agricultural commodities in the world. Mustard is widely cultivated in India, China, Eastern Europe, and Russia, with India being a major producer. India accounts for 19.8 percent of the area and 9.8 percent production of rapeseed-mustard in the world (http://www.drmr.res.in/about_rmcrop.php). Although mustard is primarily grown for edible oil and oilcakes, there is growing interest in its cultivation as a biofuel and industrial crop^1–3^.

Crop yield is the main breeding objective in mustard, but this poses challenges due to the interplay of numerous genetic and environmental factors^1,4^. Research highlights that silique per plant, seeds per silique, and seed size have the most significant direct effects on crop yield^4–6^. In *Brassica* species, an evolutionary trade-off exists between seed mass (size), seed number, and silique development^7–10^. However, focusing on individual yield components—such as silique length, rapid seed development, and bold seed size—can lead to faster selection gains, as these traits are more stably inherited and less affected by environmental changes^11–14^. Silique length and rapid seed development are key indicators of yield potential^15–18^, while bold seed size is valued for seedling establishment^19–21^. Studies in crop Brassica show that over 70% of the total photosynthetic contribution to seed growth and oil accumulation in crop Brassica comes from silique walls^22–27^. Experiments involving defoliation and shading in *B. napus* and *B. juncea* reveal that leaf photosynthesis greatly influences silique number. Additionally, silique walls serve as vehicles for seed dispersal under natural conditions through a finely regulated silique-shattering mechanism. However, uncontrolled silique shattering can adversely affect yields in managed agriculture, making resistance to premature pod shattering a vital trait for securing productivity. Resistance to premature silique shattering was possibly the earliest and most important trait selected during crop domestication^8,28,29^. A reduction in seed shattering was favoured over complete non-shattering for the ease of threshing^30,31^. Genome-Wide Association Studies (GWAS) have allowed leveraging of high-density SNP markers to identify genetic variations linked to key traits like yield, oil content, disease resistance, and stress tolerance etc^32^. Such studies have also helped to identify genetic regions affecting pod length and seed size and pod shattering in *Brassica* species. Genes like *BEE1*, PEROXIDASE, *TCP8*, *ADPG1*, *SHP1*, and *MYB116* linked to pod shatter resistance^8–33^. Regional association mapping, focussed on specific genomic regions identified using QTL mapping, has been frequently to conduct higher resolution of causal genes in rice, wheat, and mustard^34^. Studies in *Arabidopsis* have shown that seed size depends on the proliferation and expansion of cells during organ development^35,36^. TRANSPARENT GWAS has been instrumental in identifying numerous loci associated with silique-related traits such as silique length, seed number per silique, and thousand seed weight. For instance, a study identified nine loci associated with silique elongation length (SEL), with significant SNPs located on the A02 chromosome, which are crucial for enhancing seed yield by influencing seed number per silique and thousand seed weight *TESTA GLABRA2* (*TTG2*), *AUXIN RESPONSE FACTOR2* (*ARF2*) and *KLUH/CYTOCHROME P450-78A5* (*CYP78A5*) are major genes which contribute to seed size development in *Arabidopsis*^36^. These mainly promote proliferation and elongation integument^37–40^. In contrast, *APETALA2* (*AP2*) reduces the seed size by suppressing cell elongation in the integument^41^. Transcriptional profiling of the silique walls at different developmental stages has underlined the contribution of transcription factors^42^. Cytokinins act downstream of *IKU* pathway to seed development^43^. These also promote the integration of epigenetic and genetic forces for endosperm development^44^ and seed size^45^. GWAS has been instrumental in identifying numerous loci associated with silique-related traits such as silique length, seed number per silique, and thousand seed weight. *INDEHISCENT (IND), ALCATRAZ (ALC), SHATTERPROOF1 (SHP1), SHATTERPROOF2 (SHP2),* and *FRUITFULL (FUL*) are important regulators of silique dehiscence in *Brassicaceae* members^46–49^. The *TCP3* gene, regulated by miR319, also plays a role in modulating silique development and shattering resistance by influencing the expression of downstream genes like *FUL*^50^.

Indian forms of *B. juncea* are resistant to premature silique shattering^51^. However, yield losses caused by uncontrolled silique shattering have been observed in newly developed F_1_ hybrids and ‘00’ genotypes. Environmental conditions, particularly aridity, have been shown to exacerbate silique shattering. This trait is also important for aridity resilience and the prevention of yield losses due to increased fragility of siliques caused by their repeated wetting during unseasonal rains. Studies on grain legumes and cowpea indicate that arid conditions increase the frequency of shattering-resistant alleles, suggesting a natural selection pressure for resilience in such environments^52,53^. Nitrogen application and planting density, also influence shattering resistance. Optimal nitrogen levels and planting densities have been reported to enhance silique characteristics, including lignin content and structural integrity, thereby improving resistance to shattering in *B. napus*^54^. Research on mustard is limited, particularly regarding the genetic basis of silique traits in response to deficiency or excess of applied nitrogen. The present investigations were conducted to unravel the genetics underlying the phenotypic variation for silique length, seeds per silique, seed size, and silique shattering in Indian mustard under differing levels of N application. Understanding their inheritance is critical to developing an efficient breeding program, as these traits directly determine crop productivity. We used an association panel comprising 92 SNP genotyped inbred lines of Indian mustard. The average distance of LD decay and low population stratification^55^ make this panel eminently suitable for enhanced mapping resolution during genome-wide association studies.

## Results

### Phenotypic Variation and correlation analysis for traits-related to silique

**Fig. 1:**
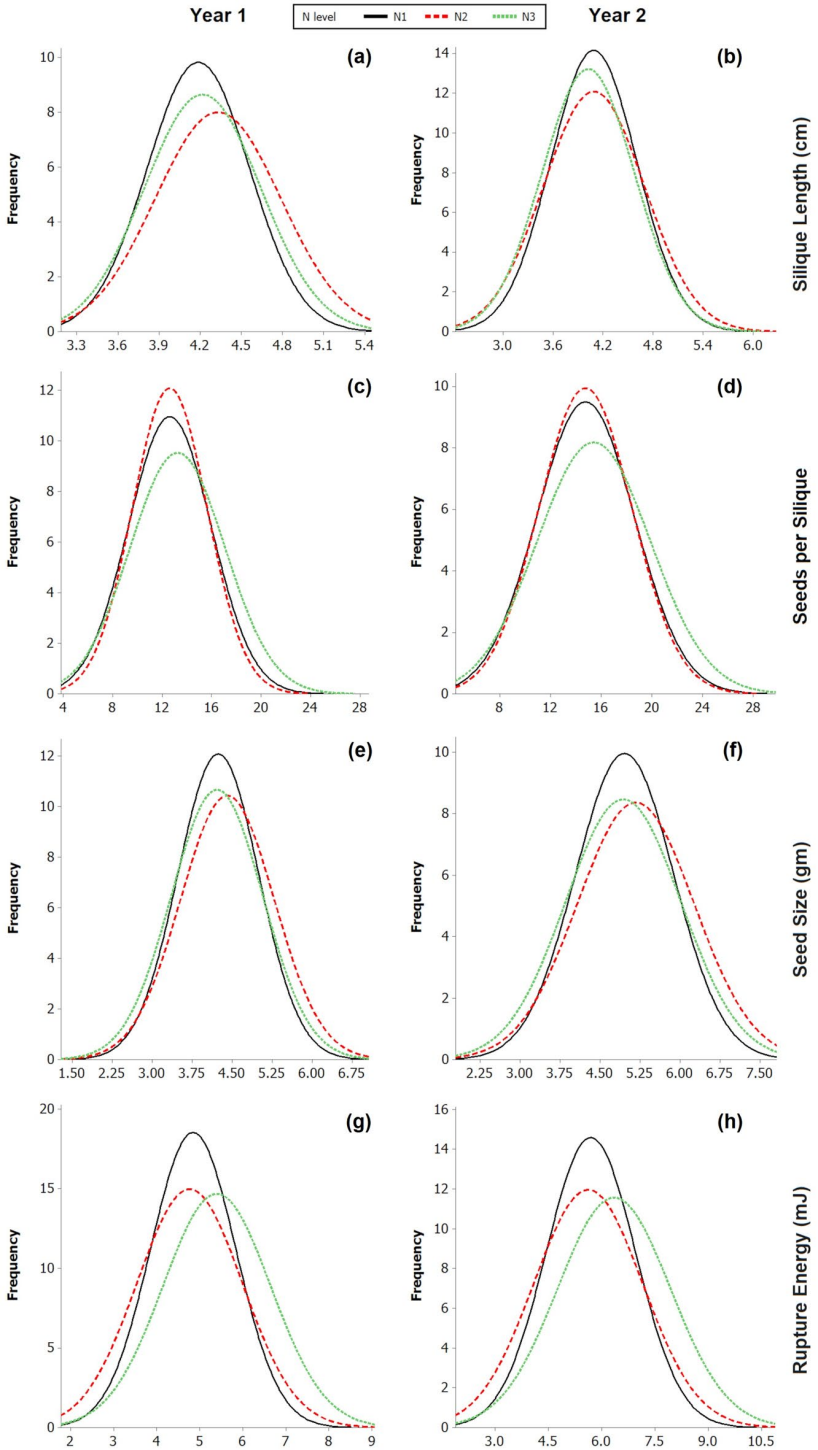
Frequency distribution curve of pod traits at three N level in 2 years (Year 1 and Year 2); (**a**,**b**) silique length, (**c**,**d**) seeds per silique, (**e**,**f**) seed size and (**g**,**h**) rupture energy required to shatter a silique in *Brassica juncea* association panel. [14.7 cm (H) × 8.4 cm (W)].

**Fig. 2:**
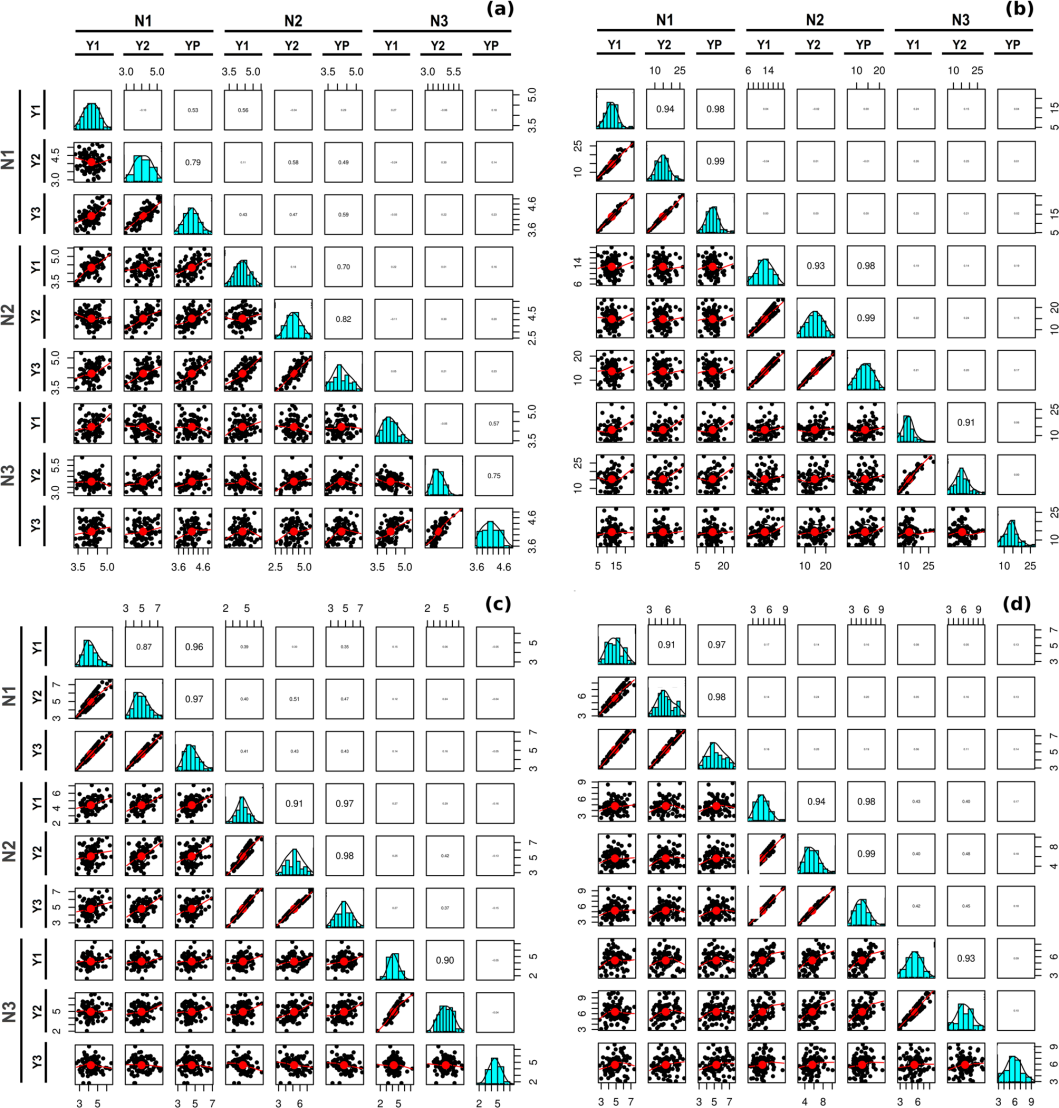
Pearson’s correlation analysis for four traits; (**a**) silique length, (**b**) seeds per silique, (**c**) seed size and (**d**) rupture energy. The correlation coefficient between years across N level are shown in upper right diagonal boxes for each traits and lower left diagonal depict the scatter plots of coefficient value. [19.8 cm (H) × 19.0 cm (W)].

The target silique attributes differed significantly at three different doses of N fertilization. The distribution of the variation was near normal (Table 1, Fig. 1). Analysis of variance (ANOVA) suggested the significance of variations due to genotypes, years, and N levels for silique length (SL), seed size (SS) and rupture energy (RE) (Table 2). SPS, SS, and RE had significant genotype x N-level interactions (GNI). However, genotypes × year interactions (GYI) were significant only for the SL. N-level × Year and Genotype × N-level × Year interactions were non-significant. Descriptive statistics for SL, SPS, SS and RE are available in Table 1. SL varied from 3.29 to 5.47 cm over all N levels during Y1. However, the trait values (2.87–6.28 cm) varied more during Y2. SL averaged at 4.14 cm for the inbred lines, with a maximum PL of 6.28 cm recorded at N3. The trait variations were at their maximum at N2 for SL. SPS ranged from 5.00 to 28.00 for Y1 and 5.88 to 28.93 during Y2. SPS averaged at 13.69 over 2 years and a maximum SPS was recorded at N3. RLM-619-AB and ELM-108 (N1), DT-70, and DT-25 (N2) and ELM-134 and ELM-151 (N3) revealed a higher number of SPS at the respective levels of N application. The mean SS was higher at N2 during both years, with a maximum of 5.17 gm during Y2. It ranged from 1.55 to 7.28 g and 2.05 to 7.68 g during Y1 and Y2, respectively. MCN-12-60 showed maximum SS at N1. RE, averaged over all genotypes, was higher in Y2 compared to Y1. The RE values ranged from 2.80 to 8.80 mJ for Y1 and 2.78 to 10.35 mJ for Y2. RE ranged from 3.05 to 9.58 mJ, when averaged over 2 years. MCP-12-606, MCN-12-40, and MCN-12-45 showed maximum RE at N1, N2, and N3 levels, respectively. Overall, SL and SS were higher at N2, whereas SPS and RE showed better performance at N3. The coefficients of variation for SL and SS were lower than those recorded for SPS and RE (Fig. 1). SPS, SS, and RE revealed highly significant correlation values (Fig. 2).

**Table 1:** Basic descriptive statistics of silique traits in *B. juncea* during 2015–16 and 2016–17.

Trait (unit)	Year	Mean ± SE			Range						Coefficient of variation		
		N1	N2	N3	N1		N2		N3		N1	N2	N3
					Min	Max	Min	Max	Min	Max			
Silique Length (SL) (cm)	Y1	4.19 ± 0.04	4.33 ± 0.05	4.22 ± 0.04	3.36	5.15	3.29	5.47	3.44	5.34	8.9	10.6	10.1
	Y2	4.09 ± 0.05	4.09 ± 0.06	4.02 ± 0.06	2.95	5.20	2.94	5.55	2.87	6.28	12.7	14.8	13.8
	YP	4.14 ± 0.03	4.21 ± 0.04	4.11 ± 0.03	3.48	4.90	3.43	5.25	3.68	4.88	7.3	10.0	7.4
Seeds per Silique (SPS)	Y1	12.61 ± 0.31	12.61 ± 0.28	13.24 ± 0.35	5.00	24.00	6.00	20.00	7.00	28.00	33.8	29.7	35.2
	Y2	14.77 ± 0.37	14.77 ± 0.34	15.42 ± 0.41	5.88	26.74	7.06	23.53	7.25	28.93	33.7	31.1	35.3
	YP	13.69 ± 2.76	13.69 ± 2.48	14.30 ± 2.77	5.44	25.37	6.53	21.77	7.12	27.05	28.7	25.7	27.5
Seed Size (SS) (g)	Y1	4.24 ± 0.07	4.41 ± 0.08	4.22 ± 0.08	2.67	6.51	2.19	7.08	1.55	7.28	23.2	24.0	25.5
	Y2	4.96 ± 0.09	5.17 ± 0.10	4.94 ± 0.10	3.11	7.47	2.81	7.68	2.05	7.61	23.7	25.9	27.4
	YP	4.60 ± 0.67	4.79 ± 0.68	4.56 ± 0.75	2.89	6.91	2.55	7.25	1.80	7.45	20.8	20.4	23.0
Rupture Energy (RE) (mJ)	Y1	4.85 ± 0.10	4.77 ± 0.12	4.22 ± 0.12	2.80	7.45	2.35	8.80	2.81	8.67	28.2	33.0	28.6
	Y2	5.70 ± 0.13	5.62 ± 0.15	6.35 ± 0.15	3.09	8.67	2.78	10.35	3.09	10.07	30.4	36.2	31.5
	YP	5.28 ± 0.98	5.20 ± 1.05	5.88 ± 1.11	3.05	7.61	2.57	9.58	3.05	9.37	28.3	28.9	27.0

**Table 2:** Analysis of variation (ANOVA) with different source of variation including interaction for silique traits.

Source of variation	DF	Mean sum of square			
		SL	SPS	SS	RE
N level	2	1.196*	31.237	6.365**	45.665**
Year	1	7.218**	1313.756**	149.007**	214.642**
Replication	1	3.108**	46.441	0.009	11.918*
Genotype	91	0.539**	73.257**	5.365**	9.954**
Genotype × Year	91	0.698**	5.857	0.644	1.027
N level × Genotype	182	0.162	48.418**	2.509**	5.341**
N level × Year	2	0.598	0.196	0.042	0.288
N level × Genotype × Year	182	0.216	0.940	0.026	0.065

*, ** and *** Significance level @ 0.05, 0.01 and 0.001, respectively.

### Genome-wide association mapping for silique traits

GWAS were conducted for four silique traits using year-wise and pooled transformed trait values. MVP facilitated the identification of significant marker trait associations (MTAs). SNPs identified as significant based on quantile–quantile (QQ) plots (Supplementary Table 1 and Supplementary Fig. S1) or environment were classified as consistent loci. Results are described separately for the traits investigated.

### Silique length (SL)

Five significant SNPs were found to be associated with SL, with the PVE ranging from 3.80 to 9.82%, on the chromosome B1, B3, B4 and B8, of which four were identified at N3 alone (Table 3; Supplementary Fig. S2). *UBIQUITIN SPECIFIC PROTEASE 15 (UBP15)*, *RING/U-box superfamily protein*, and *CONSTANS (CO)* were predicted on chromosomes B03 (N1Y1), B04 (N3Y1), and B08 (N3Y1) respectively. *MYB Domain 5 MYB5* was envisioned on chromosome B01 for the data pooled over years.

**Table 3:** Identified predicted genes and functions by GWAS and RAM analysis at N level and across the year for silique traits.

Predicted gene	Trait	N Level			CHR	GWAS				RAM			Gene function
		N1	N2	N3		SNP position/ID	No. SNPs	R^2^	Nearest SNP (kb)	SNP interval	No. SNPs	Nearest SNP (kb)	
*AT3G13540—myb domain protein 5—MYB5*	SL	–	–	YP	B01	41678091-95	2	3.80	36.80	41639724-41641762	5	0.24	Formation of the seed coat and underlying endosperm layers
*AT1G17110—UBIQUITIN-SPECIFIC PROTEASE 15—UBP15*		Y1	–	–	B03	2808011	1	9.82	47.79	2803305-2813913	4	41.89	Regulates the seed size
*AT1G17145—RING/U-box superfamily protein*		–	–	Y1	B04	19453048	1	7.81	44.82	19407232-19412373	8	0.74	Regulate Seed and Organ Size
AT5G15840—CONSTANS—CO*		–	–	Y1	B08	9746772	1	9.06	49.17	9704021-9704079	4	0.19	Regulates flowering time
*AT2G42830—SHATTERPROOF2—SHP2*	SPP, RE	Y2	–	Y1, Y2, YP	A05, B01	1402422-1412718; 3015615	12	18.11	650, 290	2063201-2063714	8	0.25	Formation of the pod
*AT4G24660—HOMEOBOX PROTEIN 22—HB22/ZINC FINGER HOMEODOMAIN 2—HB22*	SPS	–	–	Y2	A08	18859189-18859190	2	8.36	19.85	18877624-18880404	9	0.08	Floral development, female gametophyte development
*AT5G12050—BIG GRAIN 1—BG1*		Y1, YP	–	–	A10	16086496-16086528	2	14.66	18.96	16086496-16103308	4	18.96	Regulate biomass, grain size and yield
*AT3G14410—Nucleotide/sugar transporter family protein*	SS	–	–	Y1, Y2, YP	A01	36029492-36029760	3	9.38–12.30	11.02	36025796-36025895	5	7.33	Nucleotide/sugar transporter family protein
AT4G39400-BRASSINOSTEROID INSENSITIVE 1—BRI1*		–	–	Y2	A06	30893051	1	8.68	8.18	30902485-30902544	4	1.26	Enhances cell elongation; promotes pollen development, chilling and freezing tolerance; controls vasculature development
AT1G21460—Bidirectional sugar transporter SWEET1*		–	Y1, YP	–	A08	22546205-22546302	20	8.93–9.28	4.97	22550690-22555561	4	0.58	Tonoplast transporter
*AT3G13980—SKI/DACH domain protein/BIG GRAIN 4—BG4*		–	–	Y1, Y2, YP	B01	41400644-41400793	5	12.98–15.18	12.71	41415171-41419580	9	0.58	Regulates grain size
*AT1G12500—Probable sugar phosphate/phosphate translocator*		Y2, YP	–	–	B03	2121316-2121431	3	3.81–5.49	12.96	2132714-2136878	16	0.34	Nucleotide-sugar transporter
AT1G77610—EamA-like transporter family protein*		Y2, YP	–	–	B06	21574550-21574718	2	8.61–7.59	12.67	21587193-21587199	2	0.02	Amino acid transporter crucial for the amino acid homeostasis of silique
AT4G18960—AGAMOUS1—AG1*	RE	Y2		Y1, Y2, YP	A01	5791617 6481112	2	7.78	84.0	5870187-5878301	4	2.22	Regulate *SHP1*and *SHP2*
AT5G60910—FRUITFULL—FUL*		–	–	Y2	B02	59332298	1	14.52	46.0	59373033-59384081	39	0.06	Expression of the valve margin identity genes (*SHP1* and *SHP2*)
*AT4G00120—INDEHISCENT—IND*		–	Y1, Y2, YP	–	B08	55015950	1	8.39	24.7	54995359-54995510	2	4.12	Pod valve margin development

*SNPs identified within the gene; CHR, Chromosome; GWAS, Genome Wide Association Studies; RAM, Regional Association Mapping; R^2^, Phenotypic variation explained.

### Seeds per Silique

We identified 16 significant SNPs associated with this trait (Table 3, Supplementary Fig. S3), explaining 8.36% to 18.11% of the observed trait variation. Among them, 11 SNPs were located on chromosome A05 (1402422 to 1412718) at N3 for Y1 and YP. Annotation of the genomic regions around peak SNPs allowed prediction of *SHATTERPROOF2* (*SHP2*), repeatedly over Y1, Y2, and YP at N1 and N3 for both association with SPS. Additionally, *HOMEOBOX PROTEIN 22* (*HB22*) was identified on chromosome A08 (N3Y2). Furthermore, *BIG GRAIN1* (*BG1*) was located on chromosome A10 (N1, Y1, YP). It was positioned 18.96 kb from the peak SNP A10-16,086,496.

### Seed size

A total of 34 SNPs were recorded for the trait, with *R*^2^ values ranging from 3.81 to 15.18% (Table 3; Supplementary Fig. S4). These included several transporter genes such as: *AT1G12500* (Probable sugar phosphate/phosphate translocator) and *AT1G77610* (EamA-like transporter family protein) were located on chromosomes B03 and B06, respectively, at N1. *AT1G21460* (Bidirectional sugar transporter *SWEET1*) was associated with a cluster of 20 SNPs (22546205-22546302) on chromosome A08. Nine SNPs were significant at the N3 level, among which three were associated with *AT3G14410* (Nucleotide/sugar transporter family protein) on chromosome A01. SNPs linked to this gene were predicted repeatedly during Y1, Y2, and YP at N3. *BRASSINOSTEROID INSENSITIVE 1* (*BRI1*) was also predicted on chromosome A06, positioned 8.18 kb away from the associated NP. *BIG GRAIN 4* (*BG4*) was linked to five SNPs on chromosome B01 (Y1, Y2, YP).

### Rupture energy

Four identified genes together explained 48.8 percent of the observed phenotypic variation (Table 3; Supplementary Fig. S5). *AT4G18960: AGAMOUS1 (AG1)* was annotated for SNPs found on chromosome A01 at N1 (Y2) and N3 (Y1, Y2, YP). *AT5G60910—FRUITFULL (FUL)* was proposed near the SNP, B02_59332298 SNPs at N3 (Y2). *AT4G00120—INDEHISCENT (IND) -*was repeatedly envisioned on chromosome B08 at N2*.* It was expected at 24.7 kb from the peak SNP B08_55015950. *SHP2* was predicted on chromosome B01.

### Gene prediction on the basis of regional association mapping

**Fig. 3:**
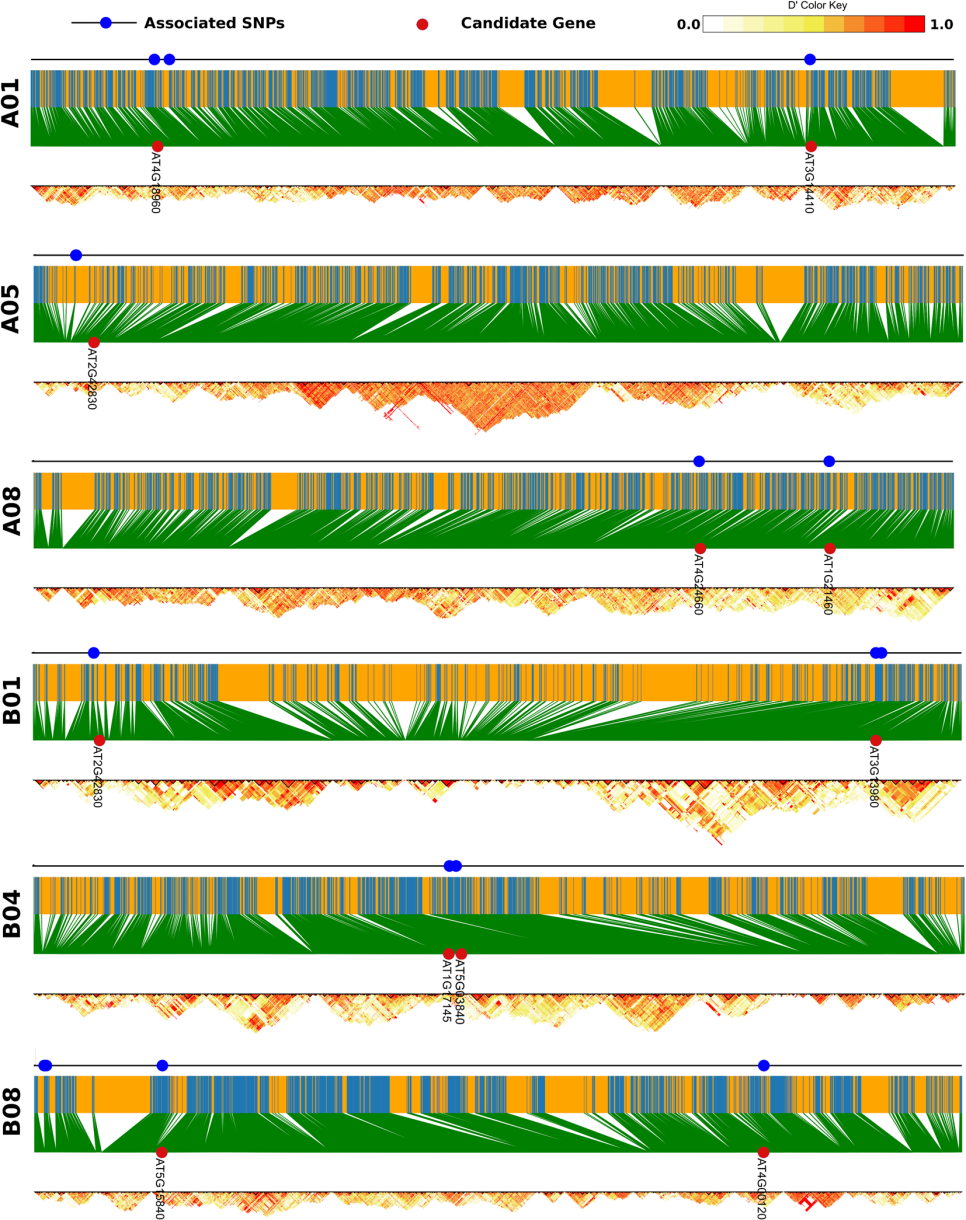
LD plot shows pairwise LD (D) estimation between SNP calculated within 1 Mb region; Honey coloured bar depict chromosome length and blue line are SNP position connected with green lines to LD region (yellow dots—low LD and red dots—high LD). Blue dots show trait associated SNPs and red dots depict the candidate gene location on the chromosome (colour figure online). [16.0 cm (H) × 12.7 cm (W)].

Regional analysis revealed a total of 136 SNPs located close to the predicted candidate genes included in the validation GWAS results (Table 3). Many of these flanked the predicted candidate genes or were present within the predicted genes (*CO, AT1G77610, SWEET1, BR1, AG1,* and *FUL*). A larger number of associated SNPs were recognised for *AT1G17145, TFL1*, *MYB5*, *HB22*, *AT1G12500,* and *BG4*. LD block estimation of predicted candidate genes on selected chromosomes fell in the region of high LD (red diagonal area) depicted in Fig. 3.

### Expression patterns of predicted candidate genes

**Fig. 4:**
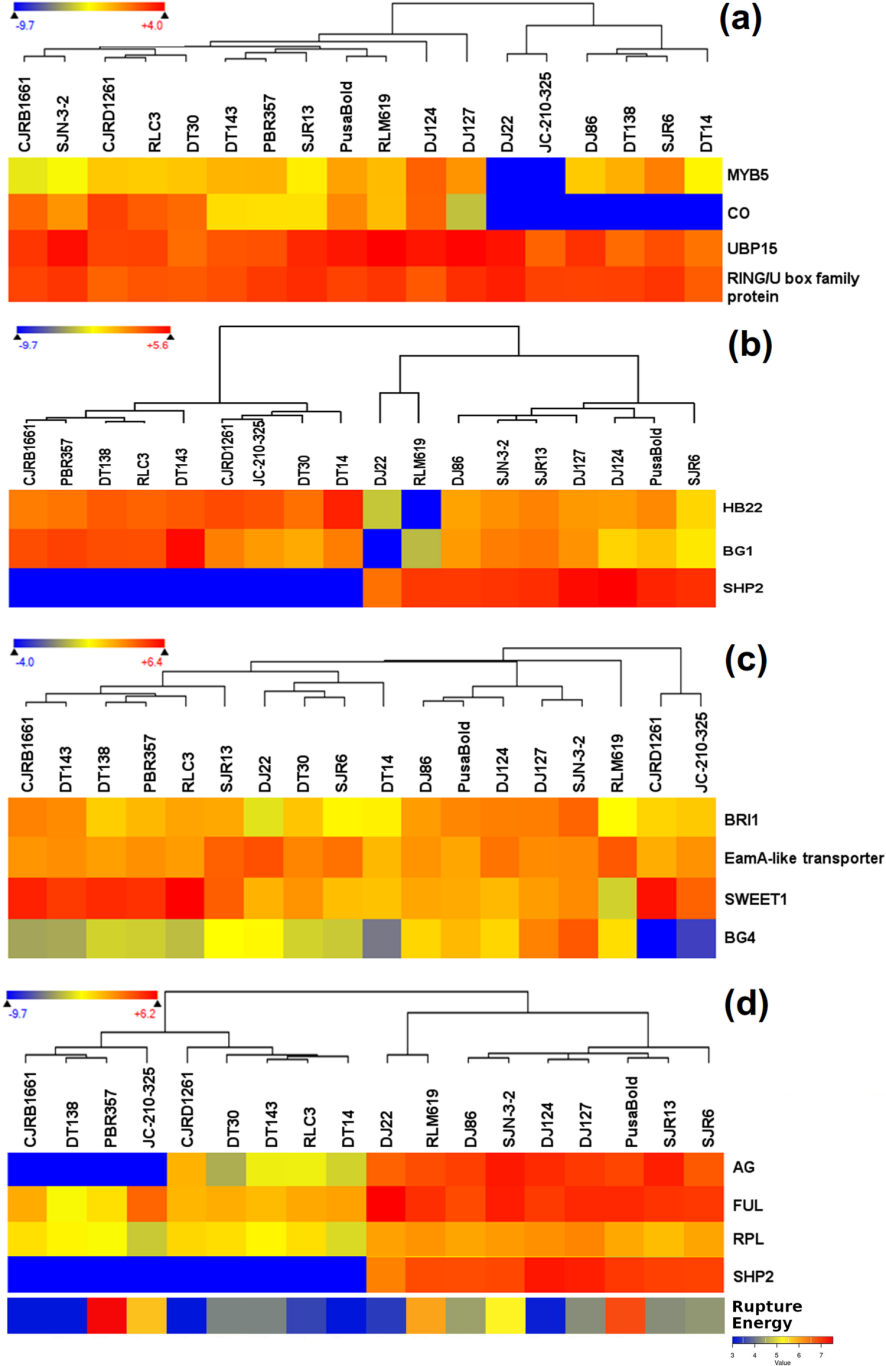
Expression level variations for the identified candidate genes**.** (**a**) Silique length, (**b**) seeds per silique, (**c**) seed size and (**d**) rupture energy. Almost all genotypes showing upregulation of *SHP2* had bold seeds. Similarly, upregulation of *AG*, *FUL* and *SHP2* was observed in the genotypes with shattering resistant genotypes. Cv. PBR-357 was the only exception to these trends. This genotype is bold seeded and possess shattering resistant silique. [38.5 cm (H) × 25 cm (W)].

RNA-seq data from 18 mustard genotypes was used to establish expression-level variations for 15 candidate genes (Fig. 4, Supplementary Table 2). Clustering based gene expression profiles produced two broad genotypic groups. However, genotypic groupings based on the gene expression profiles of some predicted candidates differed from the grouping based on their phenotypic performance. These included the upregulation of *SHP2* for SPS and the upregulation of *AG1, FUL,* and *SHP2* with resistance to silique shattering. Downregulation of *BG4* was indicated for two genotypes (CJRD1261 and JC-210-325) with the smallest seeds.

## Discussion

Crop performance in mustard depends on the number of siliques per plant. They not only house the developing seeds but also influence canopy architecture, light interception, photosynthetic potential, and the source strength of plants. Available studies shows that optimizing silique number and seed size is crucial for improving Brassica productivity. Genetic analyses have identified several QTLs associated with silique traits, particularly in rapeseed-mustard crops. However, many previous studies focused on *B. napus*, limiting their direct applicability to *B. juncea*. GWAS approaches have significantly expanded genetic information regarding silique size, seed size, and silique dehiscence. Several candidate genes, including *UBP15*, CO, SHP1, SHP2, BG1, BG4, BRI, AT1G12500, AT1G77610, SWEET1, and AT3G14410, have been identified for their roles in silique formation, grain development, and nutrient transport. Additionally, FUL, IND, and AG1 play key roles in silique shattering and seed dispersal.

Crop performance in mustard is directly associated with the number of siliques per plant, which influence canopy architecture, light interception, photosynthetic potential, and source strength. Maximum silique area index nearly equals leaf area index at the start of ripening^16^. Silique growth ends when a genetically primed seed size is achieved, which is critical for crop stand establishment under rainfed conditions. Large seeds produce seedlings with higher dry weight, root length, and root to shoot ratio compared to those raised from small seeds^56^. Seed size is also a key to cultivar acceptance by growers and processors, as bold seeds are preferred by the trade in India. Optimizing silique number and seed size is essential for improving *Brassica* productivity. Genetic analyses have produced conflicting genetic models to explain the inheritance of silique traits. These include identifying quantitative trait loci (QTLs) for silique traits in rapeseed-mustard crops^57–61^ and characterizing co-localizing QTL relationships among them^62,63^. The association panel used for current studies exhibited wide phenotypic variations and continuous distribution for all silique-related traits, underscoring their quantitative inheritance. A significant correlation between test traits is expected for traits sharing a common metabolic pool. Seeds per silique and seed size are negatively correlated, requiring a trade-off between number and size. Changes in growth conditions influence genetic variance within traits, which in turn affects genetic interactions among traits sharing a common developmental pathway.

However, most of the previous studies focused on *B. napus*, limiting their direct applicability to *B. juncea*^62^. The association panel used for current studies exhibited wide phenotypic variations and continuous distribution for all silique-related traits, underscoring their quantitative inheritance. We assessed our association panel under varying nitrogen (N) fertilization levels, recognizing that different test environments significantly influence the estimation of genetic parameters. These environmental variations play a crucial role in shaping phenotypic expression, affecting trait heritability and genetic interactions. Studies have shown that nitrogen availability impacts yield-related traits, genetic polygenicity, and selection patterns in many crops, including *B. juncea*^64^.

Nitrogen (N) is not only a vital plant nutrient but it is also a key signaling molecule, influencing various physiological and developmental processes^65^. A strong correlation among these traits was expected, given their shared metabolic pathways. Seeds per silique and seed size exhibit a negative correlation, reflecting an evolutionary trade-off between number and size. This relationship is commonly observed across plant species, where resource allocation dictates a balance between seed quantity and individual seed robustness. Marginal variations in genetic relatedness were recorded across three levels of N fertilization, highlighting the impact of environmental conditions on genetic variance. These changes further influence genetic interactions among traits that share developmental pathways, reinforcing the complexity of trait inheritance and expression.

GWAS allowed the prediction of several candidate genes related broadly to sugar transport, shoot apical meristem, floral and seed development. Important genes namely, *UBP15* and *CO* were identified for their roles in the inheritance of silique length. *CO* also functions as a metabolic switch that influences seed size and endosperm cellularization through *ABSCISIC ACID INSENSITIVE 5* (*ABI5*)^66^. *CO* further promotes flowering at the shoot apex^67^. Seed formation is a crucial reproductive process, and MADS-box genes *SHATTERPROOF 1* and 2 (*SHP1* and *SHP2*) specify integument identity in *Arabidopsis* ovules. Our studies highlighted the importance of genes associated with silique formation (*SHP2*), grain development (*BG1 and BG4*), cell elongation (*BRI*), and grain filling (*AT1G12500***,** *AT1G77610***,** *SWEET1***,** and *AT3G14410*) in determining silique traits. These genes were mapped to chromosomes in both the A (01, 06, 08) and B (01, 03, 06) genomes. Many of our findings align with previous reports. *BG1* and *BG4*, auxin-regulated genes, positively influence biomass, grain size, and yield in rice^68^. *UBP15* and *AT1G17145* regulate seed size by modulating cell proliferation in the ovule integument^69,70^. *MYB5* plays a role in trichome development and seed coat formation^71^. *BRI*, predicted on chromosome A06 in *B. juncea,* enhances cell elongation in *Arabidopsis*^72^. Mustard seeds are energy rich and store many essential nutrients, including carbohydrates, proteins, and oils, which are crucial for seed development and plant growth. Transporters facilitate nutrient import pathways, ensuring efficient allocation to developing seeds. We could identify several transporter genes, including, each playing a role in sugar and nutrient translocation. *SWEETs*, are involved in sucrose transporte from leaves to storage organs which are vital for phloem loading and source-to-sink transport^73–75^. *AT1G77610* (B06) has been implicated in seed size regulation, controlling bidirectional amino acid translocation and inorganic nitrogen allocation to maintain amino acid homeostasis in ovules and siliques^76^. Previous studies, including those by^77^, have reported small-effect QTLs on LG1, LG6, and LG13 in *B. juncea*. Additionally, past research has identified QTLs for seed number and size on chromosomes A02^6,61,78^ and A09^61,79,80^, reinforcing the complexity of genetic control over silique traits. Despite these findings, many identified QTLs and predicted gene candidates explain only a fraction of observed variation, suggesting the involvement of additional genetic factors. Current GWAS underlined the importance of *SHP2*, *IND*, *AG1,* and *FUL* in regulating silique shattering, Barring *AG1,* all genes contributing to silique dehiscence were predicted on B-genome chromosomes. *SHP1* and *SHP2* and *FUL MADS*—box genes control the development of dehiscence zone and valve margins in the siliques. *FUL* is important for silique morphogenesis. According to our results, *SHP2* is also important for determining the number of seeds per silique aside controlling the energy required to rupture a silique. *SHP2* has been shown to regulate differentiation of separation layer and lignification of the silique margins^81^. General upregulation of *AG1*, *FUL*, and SHP2 was recorded in mustard genotypes with difficult to shatter siliques. In contrast, *SHP2* was downregulated in most shattering prone mustard genotypes. *FUL* controls valve elongation and differentiation of siliques in *Arabidopsis*^46,81^. Transgenics with constitutive expression of *FUL* fail to shatter as dehiscence zone failed to form in siliques of transgenic plants^46^. *AG1* was not implicated in its role in silique dehiscence. However, *SHP1* and *SHP2* are *AG1* paralogs^82^. *AG1* and *SHP2* produce non-shatter pods if they are effectively mutated. *IND*, was also indicated at N2 over the crop seasons for the rupture energy trait. It controls the lignification of margin cells through localised depletion of auxin at the valve margins^83^. Homologs of *SHP1/2*, *FUL*, *ADPG1*, *NST1/3* and *IND* were also reported to associated with shattering in *B. juncea* and *B. napus*^84^.

Summarizing, our studies have significantly expanded genetic information regarding silique size, seed size, and silique dehiscence. Several candidate genes, including *UBP15*, *CO*, *SHP1*, *SHP2*, *BG1*, *BG4*, *BRI*, *AT1G12500*, *AT1G77610*, *SWEET1*, and *AT3G14410*, have been identified for their roles in silique formation, grain development, and nutrient transport. Additionally, *SHP2*, *FUL***,** *IND***,** and *AG1* were found to play key roles in silique shattering and seed dispersal.

## Materials and methods

### Plant material

The present studies involved field testing of 92 genotypes (comprising the *B. juncea* association panel being maintained at Punjab Agricultural University, Ludhiana. The set included Indian, Australian, Chinese, and east European germplasm lines. Details of the association panel are available at https://static-content.springer.com/esm/art%3A10.1007%2Fs11103-020-01076-x/MediaObjects/11103_2020_1076_MOESM1_ESM.pdf. These genotypes are being maintained by the Punjab Agricultural University.

### Field evaluation

Field experiments were planted according to an alpha-lattice design with three doses of nitrogen (N) fertilization. The experiment was replicated twice and repeated for two crop years [designated as Y1 (2015–16) and Y2 (2016–17), respectively] in the farms of Punjab Agricultural University, Ludhiana. The nitrogen (N) doses used were low (N1: 75 kg/ha nitrogen application), recommended (N2: 100 kg/ha nitrogen application), and high (N3: 125 kg/ha nitrogen application). Nitrogen fertiliser was applied in the form of urea (46% N), half after pre-sowing irrigation and the remaining half, 22 days after crop germination. Each germplasm line was raised in four rows, each two metres long per replication. Standard agronomic recommendations were followed for remaining crop inputs^85^. Germplasm lines were assessed for silique length (cm) and seeds per silique, seed size, and silique strength measured as rupture energy required to break open a ripe, dry silique.

### Seeds per silique (SPS)

Seeds from the 25 siliques from each of the 92 genotypes comprising the *B. juncea* association panel were collected, counted, and averaged to obtain an estimate of seeds per silique.

### Seed size (SS)

Weight of 1000 seeds was obtained in grams.

### Silique length (SL) and rupture energy (RE) needed to shatter a Silique

Silique length and rupture energy required to shatter a pod were estimated with a pendulum machine^86,87^. Rupture energy was used as a measure of shattering resistance. Five physiologically mature siliques per plant were sampled for the experiments. These were detached from the centre of the main racemes of five plants per replication. These Pods (25/germplasm line/replication) were later stored at room temperature in plastic tubes supplemented with coarse silica granules to equilibrate their moisture content. Siliques were then oven-dried at 70 °C for 24 h, before recording silique length and rupture energy (mJ) as described earlier^8^.

### Statistical analysis

An analysis of variance (ANOVA) was conducted to infer variation due to genotypes, nitrogen levels, and crop years along with their interactions. GLM (generalized linear model) was applied in alpha-lattice design using SAS software version 9.4. Correlations between levels of nitrogen and across the years of each trait were analysed and visualised using the R-package "Performance Analytics”.

### SNP genotyping

We performed genotyping by sequencing^88^ to identify single-nucleotide polymorphisms (SNPs) for association analysis. Experimental protocols used for isolating DNA, genotyping by sequencing (GBS), quality control, SNP identification, and imputation are available elsewhere^55,89^. Software ‘GAPIT’^90^ was used to develop the numerical format of SNP genotypes.

### Genome-wide association mapping (GWAS)

The Johnson transformation, as implemented in Minitab v16.0, was used to normalise phenotypic data, whenever required. An arbitrary threshold (−log10(P) > 3) was used to declare the significance of SNP-trait associations. GWAS was performed using MVP (Memory-efficient Visualization-enhanced and Parallel-accelerated Tool) (https://github.com/XiaoleiLiuBio/MVP). GLM (General Linear Model), MLM (Mixed Linear Model), and FarmCPU models were applied with principal components (PCs) as covariates. Manhattan plots were drawn with the MVP tool.

### In silico analysis to identify trait-associated candidate genes and LD blocks

Genomic regions (50 kb), upstream or downstream of the apex SNPs, were annotated to envision trait-associated candidate genes. Using A. thaliana gene models, the software Blast2GO Pro^91^ was used. The genes associated with traits of interest were annotated close to the significant SNPs present on chromosomes A01, A05, A08, B01, B04, and B05. Thus, these genes were selected for association with LD blocks using “LDblockShow” (https://github.com/BGI-shenzhen/LDBlockShow).

### Candidate gene based regional association mapping

We used an enhanced SNP set for regional association mapping (RAM) to identify SNPs closer to the gene(s) predicted from association analysis. This enhanced SNP set was developed through high-density imputation using a specifically designed mustard imputation reference panel^55,89^. SNPs with *P* values above a statistical threshold [− log10 (*P*) > 2] were rated as significant.

### Gene expression analysis

Gene expression assays were conducted to validate predicted genes, using a combined analysis of transcriptome sequences available from 18 *B. juncea* genotypes (RLM-619, Pusa Bold, RLC3, PBR357, DJ22, DJ86, DJ124, DJ127, SJN-3-2, SJR6, SJR13, DT14, DT30, DT138, DT143, CJRB-1661, CJRD1261, JC-210-325, and JC-210-325). This germplasm set was selected to reflect the germplasm variation for silique traits. RNA was extracted from young leaves and siliques harvested from plants raised under a controlled environment (16 h light; 24 °C or 18 °C day and night temperatures; 80% relative humidity) in a Conviron growth chamber. RNA extraction and shotgun sequencing were outsourced. For reference-guided transcriptome assembly, DNASTAR software by Lasergene with default parameters (RNA-Seq®. Version 15.1. DNASTAR. Madison, WI) was used. The assembled genome sequences were functionally annotated using the Blast2GO Pro pipeline against *A. thaliana* non-redundant protein database from NCBI. Differential expression analysis of predicted genes was performed with the software edgeR 2.4.3^92^. RPKM values (reads per kilobase per million mapped reads) were used to compare gene expression variation for selected set of genes.

## Electronic supplementary material

Below is the link to the electronic supplementary material.

Supplementary Material 1

## Data Availability

Short sequencing datasets have been deposited at the Sequence Read Archive (SRA) of NCBI under submission accession number PRJNA639209. The datasets used and/or analysed during the current study are available from the corresponding author upon reasonable request. The supply of germplasm resources included in the diversity set will require the approval of the Biodiversity Authority of India.
