# Supplementary material for: Genetic analysis of silique and seed traits in Brassica juncea (L.) Czern. under differential doses of nitrogen application
Source: Sci Rep. 2025 Jul 4;15:23977. doi: 10.1038/s41598-025-07758-0 (PMC12227704; doi:10.1038/s41598-025-07758-0)
Supplement: Supplementary file 1 — Supplementary Material 1 [file 41598_2025_7758_MOESM1_ESM.pdf]

Genes underlaying variation for silique and seed traits as influenced by different levels of nitrogen application in Indian mustard (*Brassica juncea* L. Czern & Coss.)

Javed Akhatar, Anna Goyal, Meenakshi Mittal, Heena, Gurpreet Kaur, Beerpal Kaur, Surinder S. Banga and Chhaya Atri *

Supplementary Table 1. Algorithm selection based on QQ-plots for identification of significant marker trait associations (MTAs).

**N level**	**N1**			**N2**			**N3**		
**Trait \ Year**	**Y1**	**Y2**	**YP**	**Y1**	**Y2**	**YP**	**Y1**	**Y2**	**YP**
**Silique Length (SL)**	FarmCPU	FarmCPU	FarmCPU	FarmCPU	FarmCPU	FarmCPU	MLM	MLM	MLM
**Seeds per Silique (SPS)**	FarmCPU	FarmCPU	FarmCPU	FarmCPU	FarmCPU	FarmCPU	MLM	FarmCPU	FarmCPU
**Seed Size (SS)**	FarmCPU	FarmCPU	FarmCPU	FarmCPU	MLM	FarmCPU	FarmCPU	MLM	MLM
**Rupture Energy (RE)**	FarmCPU	MLM	FarmCPU	FarmCPU	MLM	MLM	MLM	FarmCPU	MLM

Supplementary Table 2. Copy number variations for the genes associated with silique traits.

S. No.	Name of Gene	Expressed copy	Other Annotated copies	Annotated copies
**Silique length**				
1	CO	B08	A10	2
2	RING/U-box superfamily protein	B04	A06	2
3	MYB5	B01	A01, A02, A05	4
4	UBP15	B03	A06, B06	3
**Seeds per pod**				
5	HB22/ZINC FINGER HOMEODOMAIN 2	A08	A01, B03, B05	4
6	SHP2	B01	A04, B06 (3)	5
7	BG1	A10	A02 (2), A03, B02, B05, B08	7
**Seed size**				
8	BRI1	A06	A01, B05	3
9	SWEET1	A08	A06, B03 (2)	4
10	UDP-GALT1	B06	A07 (2), B03	4
11	BG4	B01	A01 (2), A05	4
**Rupture Energy**				
12	AG	A01	B02, B05, B07	4
13	RPL	B08	A02, A03, A10, B02, B05	6
14	SHP2	B01	A04, B06 (3 copies)	5
15	FUL	B02	A03 (2), B01, B04, B07	6

Genes underlaying variation for silique and seed traits as influenced by different levels of nitrogen application in Indian mustard (*Brassica juncea* L. Czern & Coss.)

Javed Akhatar, Anna Goyal, Meenakshi Mittal, Heena, Gurpreet Kaur, Beerpal Kaur, Surinder S. Banga and Chhaya Atri *

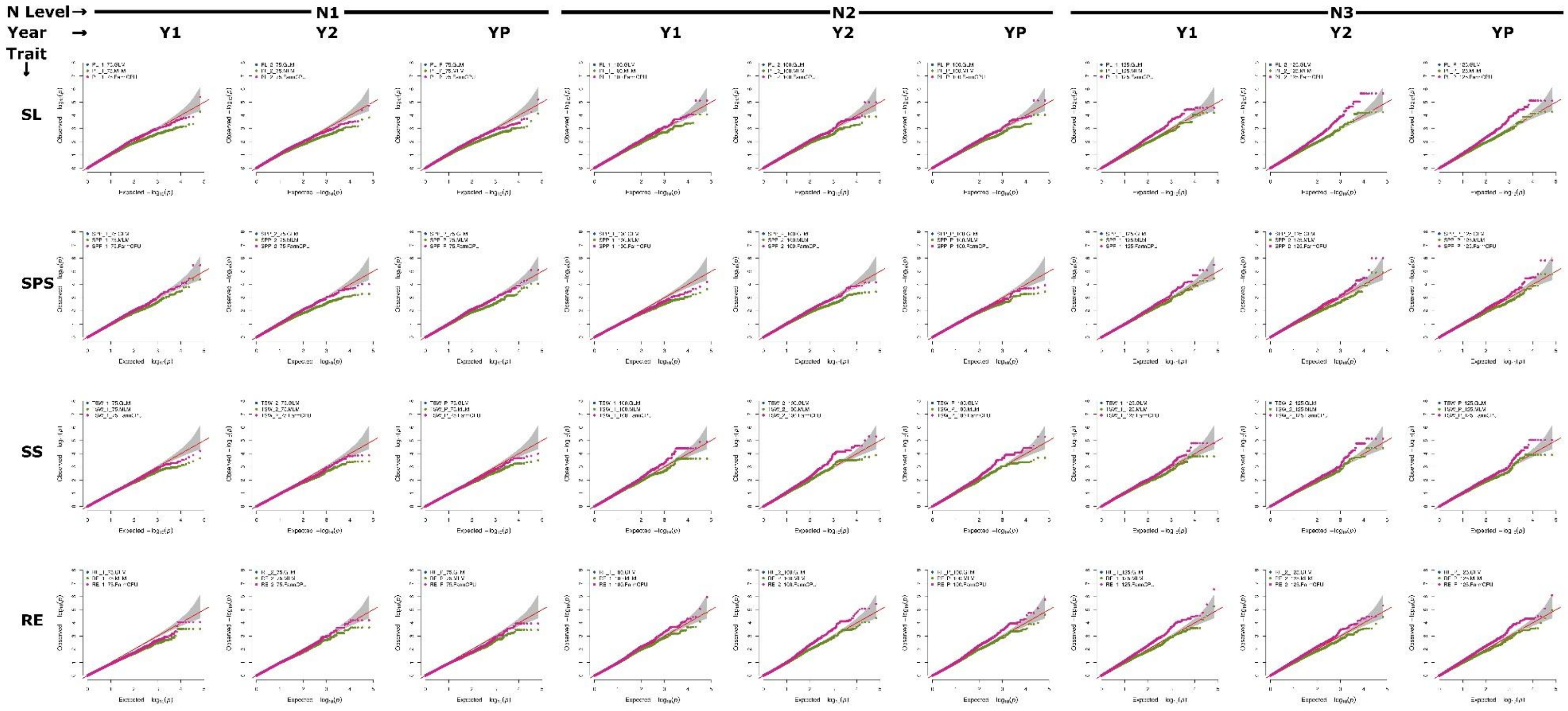

Supplementary Figure 1. Multi-model QQ-Plot for all traits at N1, N2 and N3 level across the year (Y1, Y2, YP).

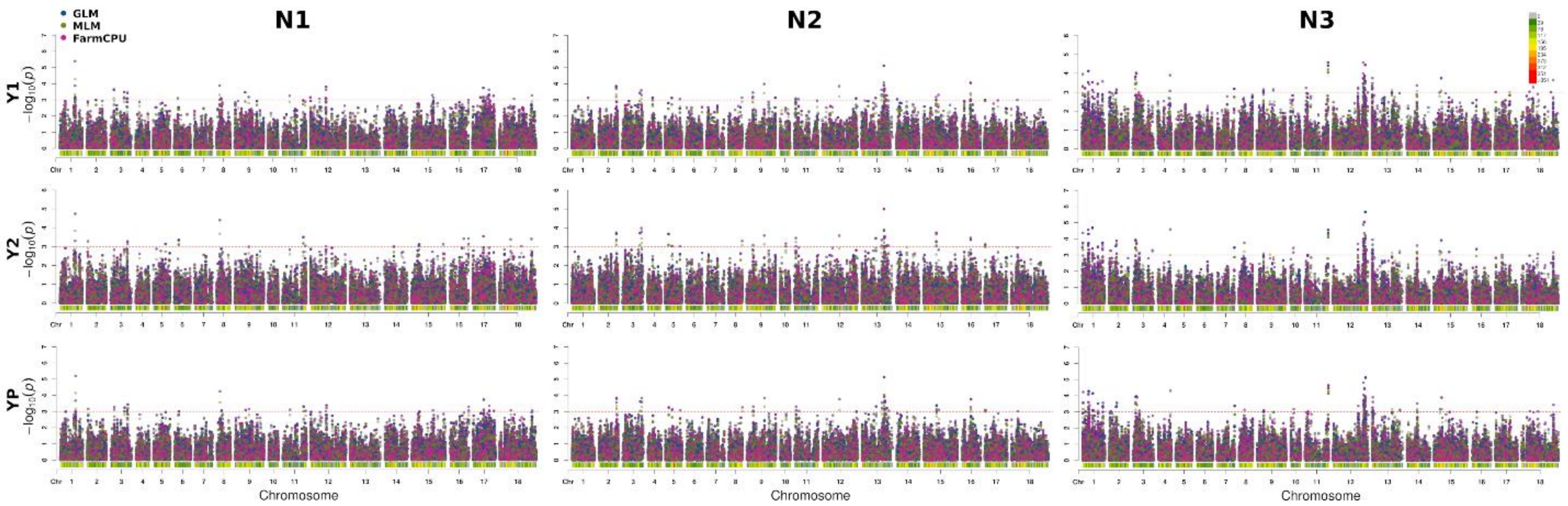

Supplementary Figure 2. Multi-model manhattan plots for Silique Length (SL) (cm) at N1, N2 and N3 level.

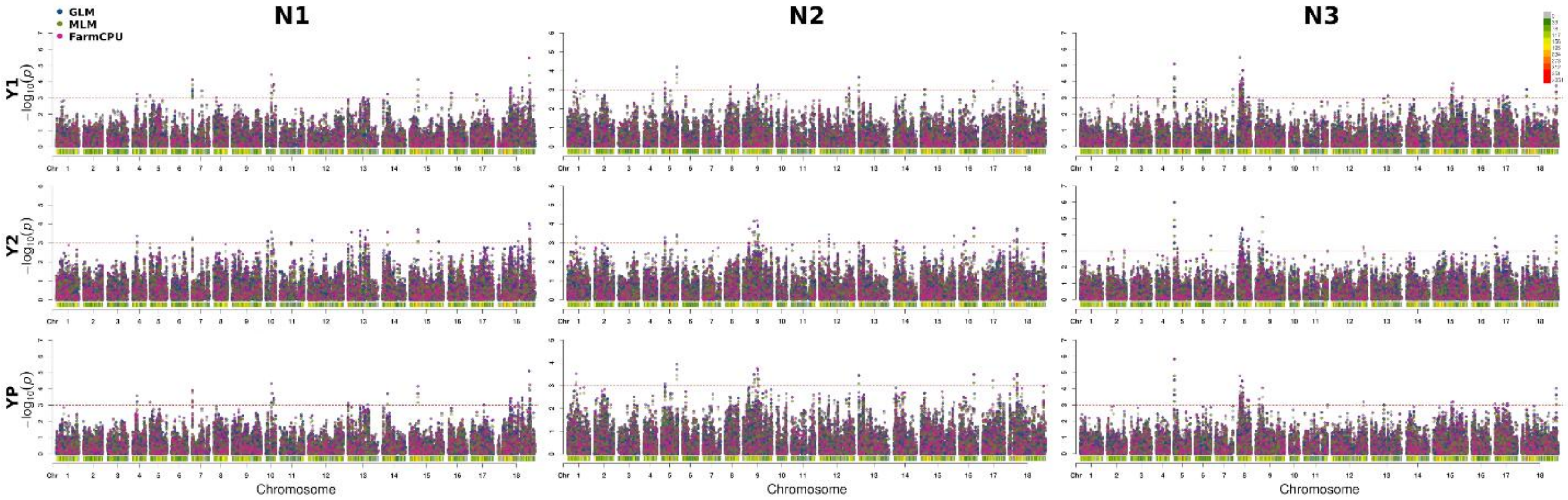

Supplementary Figure 3. Multi-model manhattan plots for Seeds per Silique (SPS) at N1, N2 and N3 level.

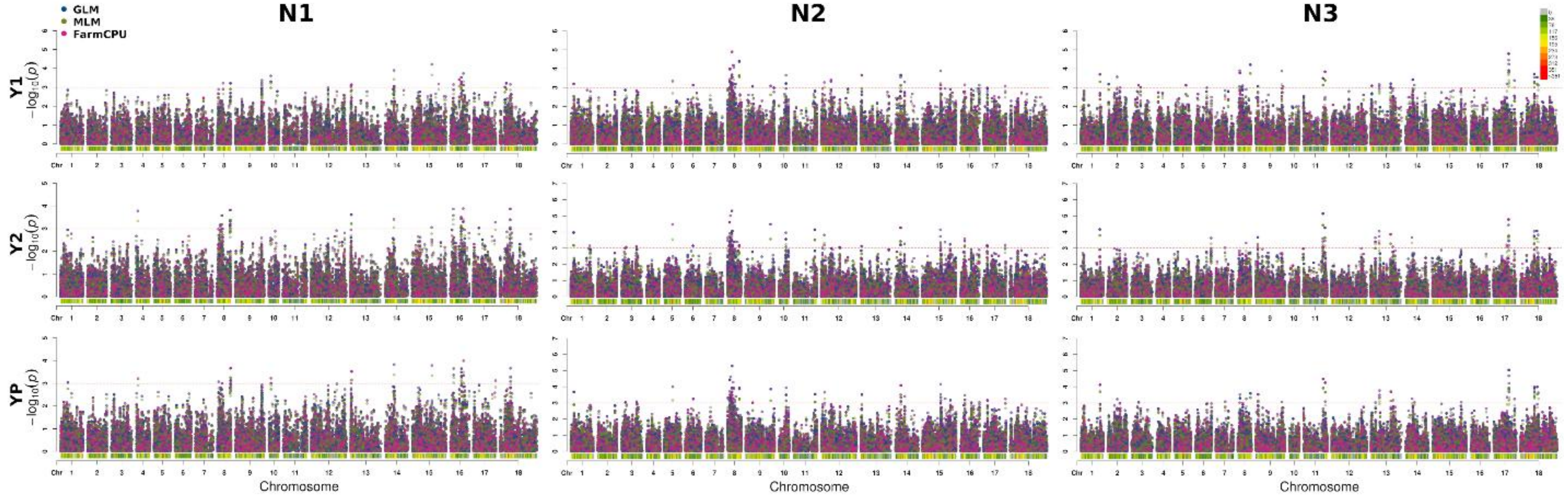

Supplementary Figure 4. Multi-model manhattan plots for Seed Size (SS) (g) at N1, N2 and N3 level.

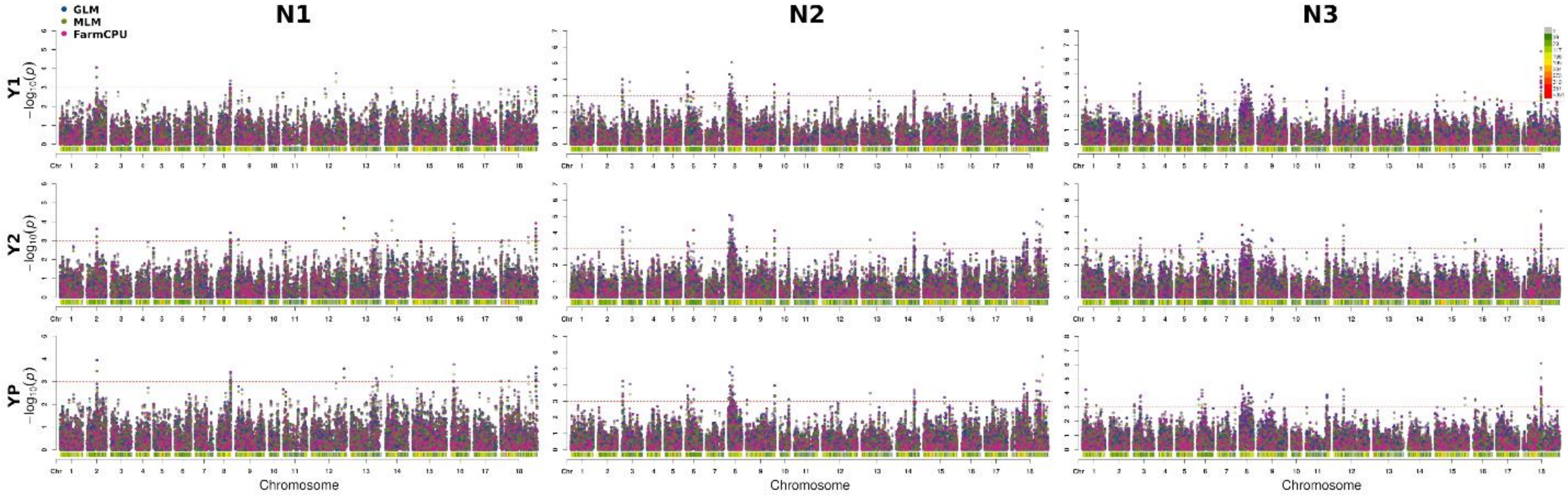

Supplementary Figure 5. Multi-model manhattan plots for Rupture Energy (RE) (mJ) at N1, N2 and N3 level
